# The Status of Soil Microbiome as Affected by the Application of Phosphorus Biofertilizer: Fertilizer Enriched with Beneficial Bacterial Strains

**DOI:** 10.3390/ijms21218003

**Published:** 2020-10-27

**Authors:** Mateusz Mącik, Agata Gryta, Lidia Sas-Paszt, Magdalena Frąc

**Affiliations:** 1 Institute of Agrophysics, Polish Academy of Sciences, Doświadczalna 4, 20-290 Lublin, Poland; m.macik@ipan.lublin.pl (M.M.); a.gryta@ipan.lublin.pl (A.G.); 2 Institute of Horticulture in Skierniewice, Pomologiczna 18, 96-100 Skierniewice, Poland; lidia.sas@inhort.pl

**Keywords:** biofertilizers, soil microorganisms, degraded soils, sustainable agriculture, biodiversity

## Abstract

Regarding the unfavourable changes in agroecosystems resulting from the excessive application of mineral fertilizers, biopreparations containing live microorganisms are gaining increasing attention. We assumed that the application of phosphorus mineral fertilizer enriched with strains of beneficial microorganisms contribute to favourable changes in enzymatic activity and in the genetic and functional diversity of microbial populations inhabiting degraded soils. Therefore, in field experiments conditions, the effects of phosphorus fertilizer enriched with bacterial strains on the status of soil microbiome in two chemically degraded soil types (Brunic Arenosol—BA and Abruptic Luvisol—AL) were investigated. The field experiments included treatments with an optimal dose of phosphorus fertilizer (without microorganisms—FC), optimal dose of phosphorus fertilizer enriched with microorganisms including *Paenibacillus polymyxa* strain CHT114AB, *Bacillus amyloliquefaciens* strain AF75BB and *Bacillus* sp. strain CZP4/4 (FA100) and a dose of phosphorus fertilizer reduced by 40% and enriched with the above-mentioned bacteria (FA60). The analyzes performed included: the determination of the activity of the soil enzymes (protease, urease, acid phosphomonoesterase, β-glucosidase), the assessment of the functional diversity of microorganisms with the application of BIOLOG^TM^ plates and the characterization of the genetic diversity of bacteria, archaea and fungi with multiplex terminal restriction fragment length polymorphism and next generation sequencing. The obtained results indicated that the application of phosphorus fertilizer enriched with microorganisms improved enzymatic activity, and the genetic and functional diversity of the soil microbial communities, however these effects were dependent on the soil type.

## 1. Introduction

Biodiversity is one of the key factors which determine the productivity and stability of ecosystems [1]. The maintenance of a high degree of variability among living organisms in terrestrial ecosystems is indisputably important due to the fact that the natural environment provides the human population with food and regulates changes to the climate [2]. From an agricultural point of view, the proper functioning of ecosystems is necessary to meet the expanding nutritional requirements of the human population [3]. In agroecosystems, particular emphasis is placed on the biodiversity of soil-inhabiting microorganisms due to their multi-faceted involvement in belowground biochemical processes. Complex communities of bacteria, fungi and archaea are responsible for the circulation of biogenic elements (*C*, *N*, *P*), organic matter decomposition and improving the status of degraded soils [4,5]. In studies focused on the symbiotic interactions between microorganisms and their host plants and the ability of soil microbiota to produce substances that adversely affect soil-borne phytopathogens, it was found that they have a positive impact on the health and development of agriculturally important plants [6]. All of these factors contribute to improved soil quality and fertility which is a crucial element in the proper functioning of agroecosystems and in the maintenance of plant production at a sufficiently high level [4].

In order to provide plants with the proper conditions for growth and development, mineral fertilizers are commonly used, which replace the nutrients present in the soil. The application of synthetic fertilizers is especially popular in regions where soils are poor in basic nutritional elements [7]. Phosphorus, along with nitrogen and potassium, is one of the most important elements in crop production, therefore traditional agriculture is heavily reliant on the intensive application of *N*, *P* and K fertilizers [8,9]. Taking into consideration the fact that phosphorus is often present in the soil in forms which are not easily accessible to plants, the application of balanced amounts of phosphorus mineral fertilizers is a determinant in the achievement of optimum yields [10,11]. Phosphorus is a vital element of compounds such as adenosine triphosphate (ATP), cytidine triphosphate (CTP), guanosine triphosphate (GTP), uridine triphosphate (UTP) and nucleic acids. It is also involved in biochemical processes responsible for the maintenance of the proper functioning of plant cells, cell division, and the activation or inactivation of intracellular enzymes and the development of the morphological structures of plants such as the roots, stalk and stem, therefore P is required at all stages of plant development [12]. Unfortunately, the non-judicious and excessive application of mineral fertilizers contributes to some undesirable effects such as lowering soil fertility and the inefficient use of substances provided with the applied agrochemicals [13]. Overloading soil with chemical fertilizers may pose a threat to the natural environment and result in the eutrophication of water resources, global warming and also in the depletion of biodiversity in agricultural soils [14,15]. Through an acknowledgement of the negative aspects of the use of traditional fertilizers, research concerning organic farming and agricultural techniques as well as substances that will contribute to efficient plant production and, at the same time, will not cause adverse changes to the environment are currently underway. Moreover, the European Green Deal policy initiatives and The EU Biodiversity Strategy for 2030 includes targets to reduce the use of fertilizers by 20% and making 25% of EU agriculture organic by the year 2030 [16]. One of the assumptions of modern agriculture is the exploitation of beneficial microorganisms which could improve the uptake of nutrients by plants and promote plant growth [17]. Biofertilizers, which contain live microbial cells, comprise an innovative, economically attractive and ecofriendly alternative to chemical fertilizers and therefore at the present time they are gaining more popularity as a tool for crop production [18]. Several bacterial species exert plant growth promoting properties and may be used as a microbial inoculants [19]. The application of bacteria from the *Bacillus* and *Paenibacillus* genera in biofertilizers is of particular importance due to their abilities including increasing the bioavailability of nutrients, boosting plant disease resistance and synthesis of plant hormones and substances directed against phytopathogens [20,21]. The phosphorus mineral fertilizer used in this experiment was enriched with strains of beneficial bacteria: *Paenibacillus polymyxa*, *Bacillus amyloliquefaciens* and *Bacillus* sp. Moreover, *Paenibacillus polymyxa* was found to exhibit phosphorus solubilization properties due to gluconic acid production and an ability for phosphonates degradation [22]. What is worth mentioning, *Paenibacillus polymyxa* is able to synthesize exopolysaccharide involved in biofilm formation which interact with plant roots and protect them from various adverse factors and take part in nutrients acquisition [23]. A study conducted by Vinci et al. [24] showed that soil inoculation with the strain of *Bacillus amyloliquefaciens* combined with compost increased the growth and nutrient exploitation of maize. It was also documented that strain of *Bacillus amyloliquefaciens* help to tolerate salt stress and can cause increase of chlorophyll content in maize seedlings [25]. Strains which belong to *Bacillus* sp. were also found to exhibit phosphorus solubilization properties through the production of organic acids and the enhancement of acid phosphatases activity [26]. Some *Bacillus* species, through the synthesis of enzymes degrading fungal cell walls (chitinase) are known to suppress *Fusarium* spp. infections in crops [27]. Taking into account the aforementioned beneficial properties of *Bacillus* and *Paenibacillus* strains, their implementation in agriculture in form of biofertilizers seems justified.

The intensification of traditional agricultural methods has led to alarming changes in agroecosystems and biofertilizers seem to be a promising approach to bridging the gap between the desire to achieve increased crop yields and the maintenance of a suitable ecological balance and a high degree of biodiversity [28]. Intensive research with biofertilizers has proven their positive impact on soil microbiological parameters and on the morphological characteristics of plants, it has been established that they pose a viable option for farmers and biofertilizers manufacturers [29]. An improved understanding of the powerful contribution of microorganisms to the agricultural production system will lead to an increase in the level of interest toward biofertilizers and the gradual reduction in the application of mineral fertilizers [30,31]. However, the development and application of conventional mineral fertilizers enriched with microorganisms is a completely new and innovative approach, which may allow for a reduction in the adverse effects of mineral fertilizers on soil environment biodiversity by decreasing the need for their use due to the addition of beneficial microorganisms to fertilizers.

Therefore, the aim of the study was to determine the immediate effects of phosphorus fertilizer enriched with beneficial bacterial strains (biofertilizer) on the status of the microbiome, biodiversity and enzymatic activity of chemically degraded soil under maize cultivation.

## 2. Results

### 2.1. Enzymatic Activity

**Figure 1:**
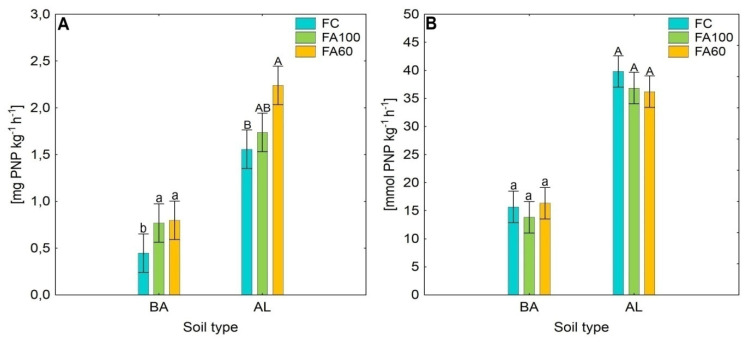

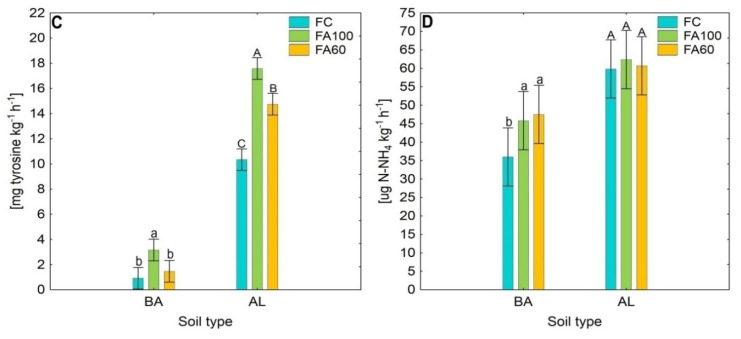
Changes in enzymes activity in soil as influenced by the application of phosphorus mineral fertilizer at an optimal dose (FC), at an optimal dose enriched with beneficial bacterial strains (FA100) and at a 40% reduced dose enriched with beneficial bacterial strains (FA60). (**A**)—the activity of β-glucosidase, (**B**)—the activity of acid phosphomonoesterase, (**C**)—the activity of protease, (**D**)—the activity of urease. Vertical bars denote 0.95 confidence intervals. Different letters indicate significant differences (*p* < 0.05). The significant differences were calculated separately for each soil type by post hoc Tukey tests after F-Welch test for β-glucosidase (**A**) and after ANOVA for the other enzymes (**B**—**D**). Different lowercase letters indicate significant differences within BA while uppercase letters within AL soil type. Explanation: BA—Brunic Arenosol, AL—Abruptic Luvisol, PNP—*p*-nitrophenol.

The application of phosphorus mineral fertilizer enriched with microorganisms contributed to changes in soil enzymatic activity in both soil types as compared to the control treatments. The activities of four soil enzymes showed various trends across the treatments (Figure 1). In general terms, the soil enzyme activities remained at a higher level in the AL soil type as opposed to the BA soil. A significant increase in β-glucosidase activity was observed in the BA soil type for FA100 and FA60 treatments as compared to the control treatment. A similar trend was observed in AL soil where β-glucosidase activity also remained at a higher level for treatments in which microbiologically enriched fertilizer was applied, with a statistically significant change occurring for the FA60 treatment. The results obtained for acid phosphomonoesterase showed that the enzyme activities remained at a similar level with no statistically significant changes across treatments for both soil types. An enhancement of protease activity was noted for the FA100 treatment in BA soil and for the FA100 and FA60 treatments in the case of AL soil. Urease activity was significantly higher for the FA100 and FA60 treatments as compared with the control for BA soil, but in the case of AL soil results showed that enzyme activities remained at a similar level for all treatments.

### 2.2. Community Level Physiological Profiles (CLPP)

#### 2.2.1. Metabolic Potential of the Soil Bacterial Community

**Figure 2:**
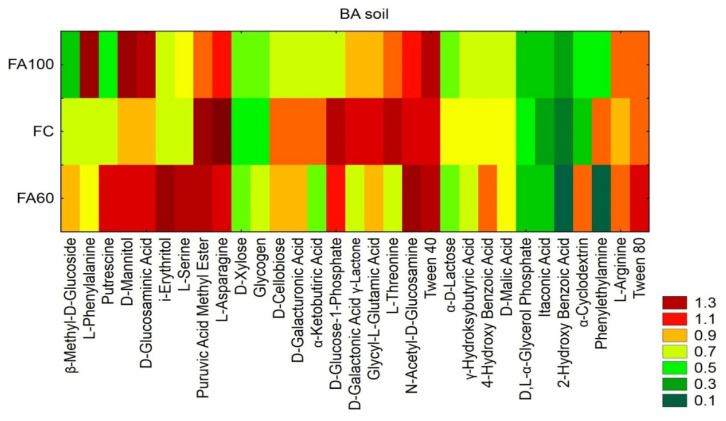

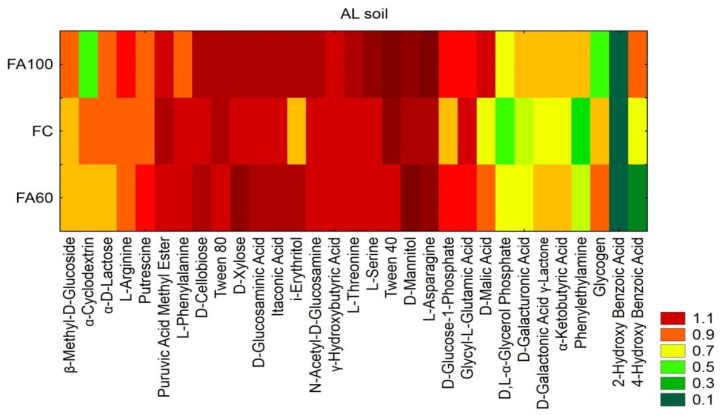
BIOLOG ECO^TM^ plate carbon substrates utilization intensity diagrams. Explanation: BA—Brunic Arenosol, AL—Abruptic Luvisol, FC—optimal dose of fertilizer, FA100—optimal dose of fertilizer enriched with microorganisms, FA60—of fertilizer enriched with microorganisms (dose reduced by 40%).

BIOLOG ECO plates were used to evaluate the capability of soil bacterial communities to utilize different carbon sources. In order to distinguish between individual C-substrates utilized to the largest and smallest extent, a multivariate statistical analysis was performed: the cluster analysis included the grouping of objects and features (Figure 2).

For BA soil, a higher degree of substrate utilization was recorded for L-phenylalanine, D-mannitol, D-glucosaminic Acid, Ls, Tween 40 and L-arginine for the FA100 and FA60 treatments as compared with the control treatment. A high rate of putrescine, i-erythritol, *N*-acetyl-D-glucosamine, 4-hydroxybenzoic Acid, α-cyclodextrin and Tween 80 utilization was also observed for the FA60 treatment. The compounds metabolized at the lowest level across all treatments were D,L-α-glycerol phosphate, itaconic acid, 2-hydroxybenzoic acid and, in case of the FA60 treatment, phenylethylamine.

For the AL soil type, out of the 31 different *C*-compounds, the increased utilization of D-cellobiose, D-xylose, D-glucosaminic acid, itaconic acid, i-erythritol, D-mannitol, L-asparagine, D-glucose-1-phosphate, D-malic acid, D,L-α-glycerol phosphate, D-galacturonic acid, D-galactonic acid γ-lactone, α-ketobutyric acid and phenylethylamine was observed for the FA100 and FA60 treatments in contrast to the FC treatment. A high utilization rate was reported for β-methyl-D-glucoside, L-arginine, L-threonine, L-serine, Tween 40 and 4-hydroxybenzoic Acid for the FA100 treatment and for putrescine and glycogen for the FA60 treatment. It was also noted that 2-hydroxy- benzoic acid was metabolized in the lowest degree between all treatments and 4-hydroxybenzoic acid in the case of FA60 treatment. The AWCD and Richness indices were higher for the FA100 and FA60 treatments as compared to the control for the AL soil type. The increase in the Shannon and Richness indices were observed for the BA soil fertilized with FA100 (Table 1).

#### 2.2.2. Growth Pattern of Soil Fungal Community on Different Carbon Sources

**Figure 3:**
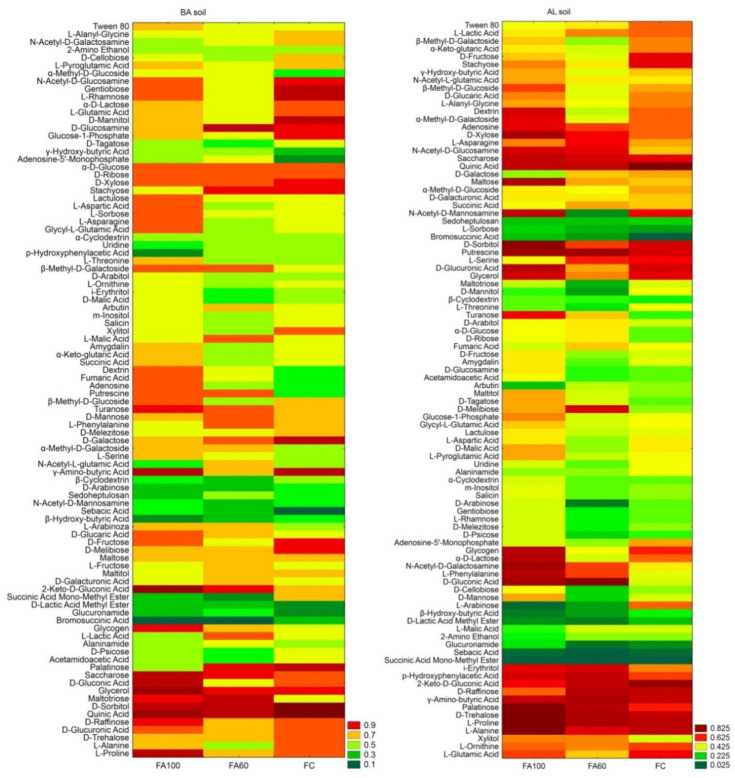
Diagrams of fungal growth intensity on the BIOLOG FF^TM^ plate carbon substrates. Explanation: BA—Brunic Arenosol, AL—Abruptic Luvisol, FC—optimal dose of fertilizer, FA100—optimal dose of fertilizer enriched with microorganisms, FA60—fertilizer enriched with microorganisms (dose reduced by 40%).

Research concerning the functional diversity of fungi allowed for the determination of the impact of biofertilizers on the growth pattern of filamentous fungi inhabiting the soil under maize cultivation. The results of a thorough assessment of fungal growth patterns as expressed by biomass production in the experimental treatments are shown in Figure 3.

In BA soil, it was found that fungal communities were able to grow on substrates such as β-methyl-D-galactoside, putrescine, turanose, L-arabinose, D-glucaric Acid, 2-keto-D-gluconic acid, glycogen, α-methyl-D-galactoside, maltotriose and saccharose at relatively high levels for the FA100 and FA60 treatments as compared to the control. A higher degree of biomass production was also recorded for *C*-compounds such as Tween 80, lactulose, L-aspartic Acid, L-sorbose, L-asparagine, glycyl-L-glutamic acid, L-threonine, dextrin, fumaric acid, adenosine, β-methyl-D-glucoside, D-gluconic acid, D-raffinose, D-glucuronic Acid, L-proline, amygdalin, α-ketoglutaric acid, succinic acid and glycerol for the FA100 treatment. In the case of the FA60 treatment, it was reported that a higher level of growth was found on substrates such as D-glucosamine, arbutin, L-malic acid, D-mannose, L-phenylalanine, *N*-acetyl-L-glutamic acid, L-fructose, D-galacturonic acid and L-lactic acid. The lowest biomass production on substrates across all treatments included sebacic acid, D-lactic acid methyl ester and bromosuccinic acid.

For AL soil, out of 95 various *C*-compounds an increased turbidity for D-xylose, *N*-acetyl-D-glucosamine, putrescine, turanose, D-melibiose, *N*-acetyl-D-galactosamine, L-phenylalanine, D-gluconic acid, i-erythritol, *p*-hydroxyphenylacetic acid, palatinose, xylitol and adenosine was observed for the FA100 and FA60 treatments in comparison with the control soil. For the FA100 treatment an increased degree of growth was noted for β-methyl-D-glucoside, dextrin, α-methyl-D-galactoside, saccharose, maltose, *N*-acetyl-D-mannosamine, D-sorbitol, D-glucuronic acid, turanose, maltitol, D-tagatose, glucose-1-phosphate, glycyl-L-glutamic acid, D-malic acid, L-pyroglutamic acid, glycogen, α-D-lactose, D-mannose, palatinose, D-trehalose, L-proline and L-alanine, and for the FA60 treatment L-asparagine and fumaric acid utilization was reported. The lowest biomass production on substrates across all treatments comprised sedoheptulosan, L-sorbose, bromosuccinic acid, D-lactic acid methyl ester, glucuronamide, sebacic acid and succinic acid monomethyl ester. Increased AWDD values were observed for FA100 treatments in both soil types. Higher Richness and Shannon indices values were reported for the FA100 and FA60 treatments in BA soil and for FA100 treatment in AL soil (Table 1).

### 2.3. Terminal Restriction Fragment Length Polymorphism

As a result of analyses, the restriction profiles of the individual taxa of microorganisms, consisting of a pattern of DNA fragments of different lengths were obtained. The restriction profiles of individual taxa differed in the length of fragments as well as their relative abundance. The differences in the number of fragments within a particular group of microorganisms between the FC, FA100 and FA60 treatments were also recorded. Within the total pool of T-RFs, there were fragments presented for all experimental treatments and those that were only characteristic of a particular fertilization method.

**Figure 4:**
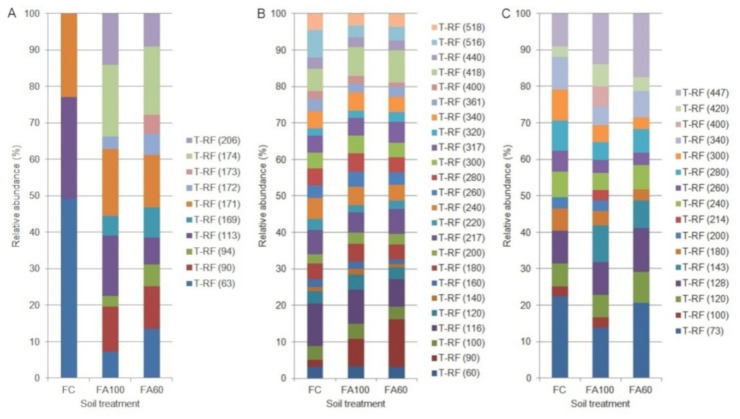
Relative abundance of the terminal restriction fragments obtained after HaeIII digestion for the bacterial (**A**), archaeal (**B**) and fungal (**C**) communities in Brunic Arenosol soil. Explanation: FC—optimal dose of fertilizer, FA100—optimal dose of fertilizer enriched with microorganisms, FA60—fertilizer enriched with microorganisms (dose reduced by 40%).

**Figure 5:**
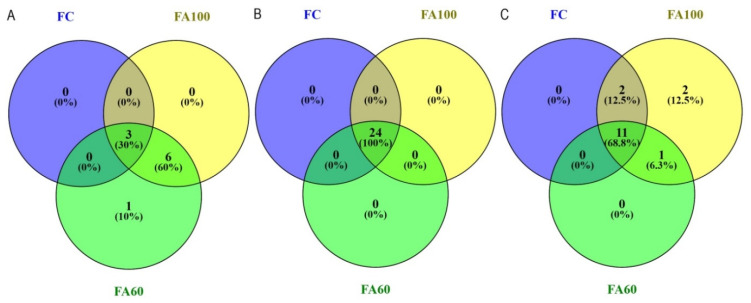
Venn diagrams showing the number of shared and unique terminal restriction fragments among bacteria (**A**), archaea (**B**) and fungi (**C**) communities in Brunic Arenosol soil. Explanation: FC—optimal dose of fertilizer, FA100—optimal dose of fertilizer enriched with microorganisms, FA60—fertilizer enriched with microorganisms (dose reduced by 40%).

The restriction profiles for a BA soil type are presented in the Figure 4. The bacterial restriction profile was characterized by the presence of 10 T-RFs of 63–206 bp, with the presence of each fragment reported in the FA60 treatment (Figure 4A). For the FC treatment, three restriction fragments were reported with a relative high abundance of 63 bp T-RF. The T-RF that only appeared in the FA60 treatment was a fragment of 173 bp. The differences in the relative abundance of 63, 113, 171 bp T-RFs across all treatments were also noted. According to the Venn diagrams, which were prepared in order to visualize which T-RFs were shared between particular treatments, 60% of the terminal restriction fragments obtained were common for treatments with applied biofertilizers and 30% of the total T-RFs number were shared across all treatments (Figure 5A).The presence of 24 restriction fragments of 60–518 bp was noted in the case of the archaeal restriction profile (Figure 4B), so it was characterized by the greatest variety of obtained T-RFs as compared with bacteria (Figure 4A) and fungi (Figure 4C). 100% of the obtained T-RFs were presented across all treatments with no specific fragments found for the individual fertilization method (Figure 5B). It was reported that the relative abundance of 90 bp T-RF increased in treatments where microbiologically enriched fertilizer was applied as compared to treatments where traditional mineral fertilizer was used. In the fungal restriction profile, 16 T-RFs of 73–447 bp were observed (Figure 4C) and 68.8% of them were common for all treatments (Figure 5C). All of the obtained T-RFs were found to be present in the FA100 treatment with the fragments of 214 and 400 bp specific for this fertilization method. The 73 bp fragment was the most abundant across all treatments. Compared to the FC, an increase in the relative abundance of the 447 bp T-RF in the FA100 and FA60 treatments was reported. The T-RF which only appeared in FA100 and FA60 was 143 bp.

The predominant bacterial T-RF 63 in the control soil which is present in treatments with biofertilizers could be represented by *Rathayibacter* and *Caldicoprobacter* (Table S1), which was assessed based on a prediction approach used in silico analysis with TRiFLe software [32] and NCBI database (https://www.ncbi.nlm.nih.gov/) [33] The archaeal T-RF 90 present in all tested treatments (FC, FA100, FA60) which increases after biofertilizer application could be a representative of *Methanocaldococcus* and *Thaumarchaeote* based on in silico analysis mentioned above. Finally, the fungi connected with 447 T-RF may belong to the genus *Aspergillus* and *Pyrenochaetopsis* (Table S1).

**Figure 6:**
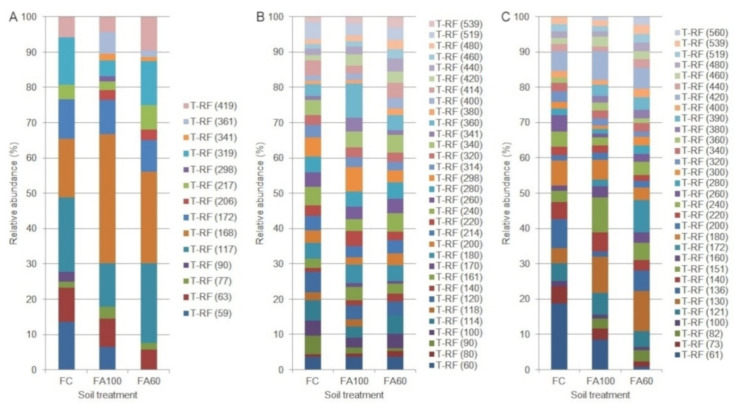
Relative abundance of the terminal restriction fragments obtained after HaeIII digestion for bacterial (**A**), archaeal (**B**) and fungal (**C**) communities in Abruptic Luvisol soil. Explanation: FC—optimal dose of fertilizer, FA100—optimal dose of fertilizer enriched with microorganisms, FA60—fertilizer enriched with microorganisms (dose reduced by 40%).

**Figure 7:**
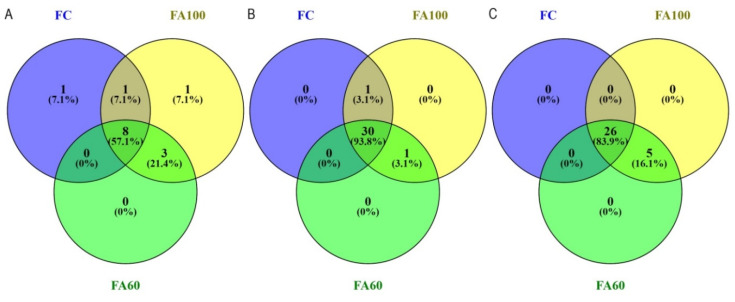
Venn diagrams showing the number of shared and unique terminal restriction fragments among bacteria (**A**), archaea (**B**) and fungi (**C**) communities in Abruptic Luvisol soil. Explanation: FC —optimal dose of fertilizer, FA100—optimal dose of fertilizer enriched with microorganisms, FA60—fertilizer enriched with microorganisms (dose reduced by 40%).

Results from the AL soil type are presented in Figure 6. For the Abruptic Luvisol soil a greater variety of restriction fragment sizes was observed in comparison with Brunic Arenosol. In the bacterial restriction profile, the presence of 14 T-RFs with a size of 59–419 bp was noted, with the greatest degree of diversity characterized by the FA100 treatment, in which 13terminal DNA fragments were found to be present (Figure 6A). Fragments unique to the treatments in which microbiologically enriched phosphorus mineral fertilizer was applied were T-RFs with the size of 206, 298, 341 and 361 bp and they constituted 28.5% of all T-RFs. In relative terms the most abundant T-RFs were 117 and 168 bp, with an increase in the abundance of the 168 bp fragment for the FA100 and FA60 treatments. It was also reported that 57.1% of the obtained T-RFs were common to all of the tested treatments (Figure 7A). In the case of archaea, a restriction profile consisting of 32 fragments of 60–539 bp (Figure 6B) was obtained and the majority (93.8%) of them were shared between all of the treatments (Figure 7B). FA100 was characterized by the greatest variety of T-RFs. For the same soil treatment, in relative terms the most abundant fragment was T-RF with a size of 360 bp. A fragment of 170 bp only appeared in the FA100 and FA60 treatments. The profile obtained for the fungal communities consisted of 31 restriction fragments with a size range of 61–560 bp (Figure 5C). All of these T-RFs were found in the FA100 and FA60 treatments, with fragments of 82, 172, 380, 390 and 560 bp with a size unique to them, which represents 16.1% of all the obtained T-RFs (Figure 7C). For the FA100 treatment an increase in the occurrence of 151 bp and 420 bp fragments was noted, while for the FA60 treatment a T-RF of 172 bp was found as compared to the control soil. An increase in the relative abundance of 130 bp T-RF in FA100 and FA60 was reported in comparison with FC. A terminal restriction fragment with a size of 61 bp was the most abundant in relative terms across the whole restriction profile.

Based on in silico analysis selected T-RFs were assigned to the following representatives of bacteria *Caloramator* (117 bp), *Pelosinus*, *Pandoraea* and *Burkholderia* (168 bp), archaea *Nitrososphaera* and *Euryarchaeotae* (360 bp) and fungi *Mucor* (200 bp), *Clonostachys* (420 bp) and *Penicillium* (82 bp) (Table S1).

Jaccard’s coefficient index, based on the presence or absence of T-RFs and their relative abundance, was used to analyse similarities between the M-tRFLP profiles of soil microbial communities. The Jaccard coefficient values range from 0 to 1, where 1 indicates that the communities are identicals and 0 indicate no connections between them [34]. In this study it was reported that Jaccard’s coefficient values were higher in bacterial and fungal profiles in both soil types between the FA100 and FA60 treatments (Table 2).

### 2.4. Next Generation Sequencing

#### 2.4.1. Alpha Diversity

**Figure 8:**
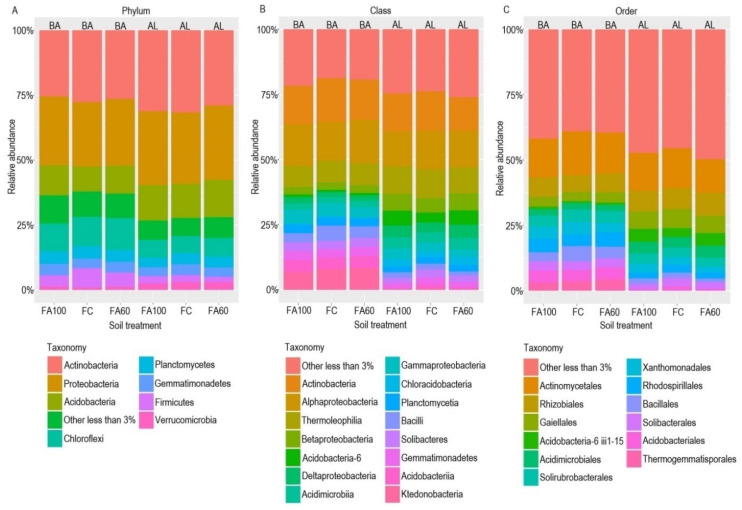
Distribution of bacterial phyla (**A**), classes (**B**) and orders (**C**) obtained by next generation sequencing of DNA extracted from soil samples. Explanation: BA—Brunic Arenosol, AL—Abruptic Luvisol, FC—optimal dose of fertilizer, FA100—optimal dose of fertilizer enriched with microorganisms, FA60—fertilizer enriched with microorganisms (dose reduced by 40%).

**Figure 9:**
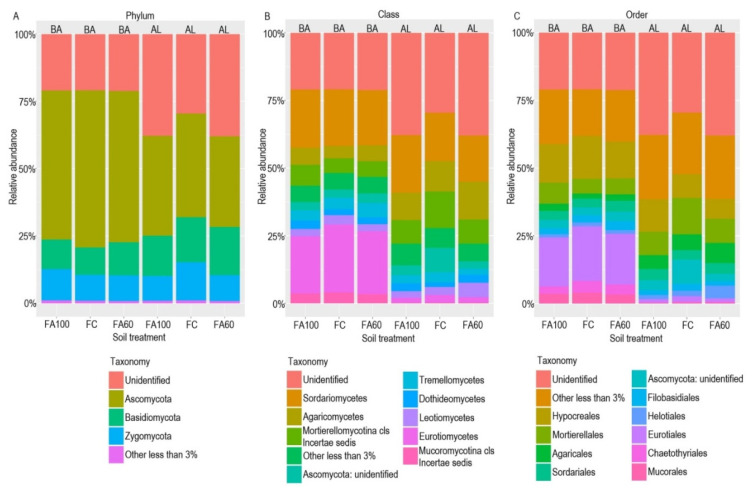
Distribution of fungal phyla (**A**), classes (**B**) and orders (**C**) obtained by next generation sequencing of DNA extracted from soil samples. Explanation: BA—Brunic Arenosol, AL—Abruptic Luvisol, FC—optimal dose of fertilizer, FA100—optimal dose of fertilizer enriched with microorganisms, FA60—fertilizer enriched with microorganisms (dose reduced by 40%).

The soil microorganism community structure from both soil types was analysed through next generation sequencing. The results of NGS indicated differences in the composition of microbiomes between particular treatments (Figure 8 and Figure 9).

The most abundant phyla among the tested soils were Actinobacteria (25.49–31.68%) and Proteobacteria (24.78–28.59%). The third and fourth most numerous phyla constituted Acidobacteria (9.60–14.39%) and Chloroflexi (6.52–12.18%), respectively (Figure 8A). It is worth noting that for the FA100 and FA60 treatments, in both soil types, the increased relative abundance of bacteria belonging to the Proteobacteria, Acidobacteria and Chloroflexi groups was reported, as compared to the control soil (FC).

The prominent classes in the bacterial metagenome in both soil types were Actinobacteria (12.81–16.79%), Alphaproteobacteria (13.46–16.59%) and Thermoleophilia (8.05–11.04%) (Figure 8B). Bacteria belonging to the Ktedonobacteria group were also characterized by a relatively high abundance (6.68–8.41%) as compared to other identified classes. In Brunic Arenosol soil it was observed that the relative abundance of Alphaproteobacteria was higher for the FA100 (15.59%) and FA60 (16.59%) treatments in comparison with FC (14.86%). A similar trend was noted in the case of Betaproteobacteria (FC-5.44%, FA100-6.41%, FA60-6.56%), Acidobacteria-6 (FC-3.87%, FA100-5.70%, FA60-5.49%) and Deltaproteobacteria (FC-3.71%, FA100-4.53%, FA60-5.06%) in Abruptic Luvisol soil.

At the taxonomic order level, it was observed that the most abundant were members of Actinomycetales (12.81–16.79%) and Rhizobiales (6.37–8.80%) for both soil types (Figure 8C). The relatively high abundance of Gaiellales (6.63–7.32%) was also reported in AL soil. In Brunic Arenosol the increased relative abundance of Rhizobiales, Gaiellales and Rhodospirillales (FC-4.48%, FA100-5.13%, FA100-5.48%) was noted for treatments where microbiologically enriched fertilizer was applied, as compared to the control soil. The same trend was observed for Acidobacteria-6 iii1-15 (FC-3.43%, FA100-4.95%, FA60-4.81%) and for Rhizobiales (FC-8.09%, FA60-8.80%) in Abruptic Luvisol soil.

The dominant bacteria for particular treatments are shown in Table S2. 16s rDNA next generation sequencing revealed the presence of Archaea in the tested soils, however, their relative abundance was low and remained between 0.03–0.09%, depending on the soil type and fertilization treatment (Table S3). The archaeal community was mainly composed of Crenarchaeota at the phylum level (0.02–0.07%). The tested soils were almost devoid of Euryarchaeota (<0.00 to 0.01%) and Parvarchaeota (<0.00%). Among Crenarchaeota two classes were identified: marine benthic group archaea (MBGA) (<0.00–0.04%) and Thaumarchaeota (0.02–0.03%). Two classes belonging to the Euryarchaeota group were also identified, namely Methanomicrobia (<0.00%) and Thermoplasmata (>0.00–0.01%). At the order level, NRP-J were identified among MBGA with a relative abundance 0.00–0.04% and Cenarchaeales and Nitrososphaerales with a percentage share of 0.00–0.01% and 0.02%, respectively. The representatives of Methanomicrobia belonging to the Methanosarcina order comprised <0.00% in the archaeal community. In the Thermoplasmata class NGS revealed the presence of the E2 order with a relative abundance of >0.00–0.01%.

The fungal community was found to be comprised mainly of three phyla, with the dominance of Ascomycota (33.70–56.15%), followed by Basidiomycota (10.17–17.90%) and Zygomycota (9.24–14.23%) (Figure 9A). A relative increased in abundance was observed for Basidiomycota for the FA100 and FA60 treatments in case of BA soil and for the FA60 treatment in AL soil. In BA soil fungi belonging to the Zygomycota group were also characterized by a higher relative abundance for the FA60 treatment as compared to the control soil.

Among all of the identified classes, for BA soil the most numerous fungi were Sordariomycetes (20.41–21.56%) and Eurotiomycetes (21.27–23.20%). Sordariomycetes were also relatively abundant in AL soil (17.07–21.34%), followed by Agaricomycetes (10.14–14.01%) and Mortierellomycotina cls Incertae sedis (8.71–13.52%) (Figure 9B). When comparing particular treatments it was reported that the relative abundance of Sordariomycetes was higher for the FA100 treatments than for FC in both tested soils (FC_BA_-20.94%, FA100_BA_–21.56%, FC_AL_-17.87%, FA100_AL_-21.34%). A similar trend was noted for the Agaricomycetes (FC_BA_-4.57%, FA100_BA_-6.23%, FA60_BA_-5.88%, FC_AL_-11.26%, FA60_AL_-14.01%). On the other hand, in the case of Mortierellomycotina cls Incertae sedis, which was one of the dominant classes in AL soil, a decrease in relative abundance was reported for treatments in which microbiologically enriched fertilizer was applied as compared to the control soil (FC_AL_-13.52%, FA100_AL_-8.71%, FA60_AL_-8.92%).

A closer examination of the taxonomy of identified fungi revealed that the dominant orders in BA soil were Hypocreales (13.66–15.84%) and Eurotiales (18.12–20.27%) (Figure 9C). Hypocreales were also quite numerous in AL soil, with the increase in their relative abundance occurring for the FA100 treatment (FC-8.73%, FA100-11.99%). The second most abundant order in AL soil was Mortierellales, whose percentage share was higher in FC (13.52%) than in FA100 (8.71%) and FA60 (8.92%) treatments. Certain fungi which had a relative increase in abundance were found in treatments with applied biofertilizers and belonged to the Agaricales group (FC_BA_-1.86%, FA100_BA_-2.64%, FA60_BA_-2.38%, FC_AL_-5.79%, FA60_AL_-7.46%) and the Sordariales group (FC_BA_-3.24%, FA100_BA_-3.41%, FA60_BA_-3.99%, FC_AL_-3.51%, FA100_AL_-4.22%, FA60_AL_-3.93%).

The dominant fungal species for particular treatments are shown in Table S4. The number of obtained OTUs are presented in Table 1. An increased number of operational taxonomic units was reported as a result of a 16S rDNA analysis in BA soil for the FA100 and FA60 treatments and also as a result of an ITS1 analysis for both soil types in treatments where microbiologically enriched fertilizer was applied as compared to the control soil. Simultaneously, higher values of the Shannon index for the FA100 and FA60 treatments were noticed. However, there were no statistically significant changes between treatments in the aforementioned indices.

#### 2.4.2. Beta Diversity

**Figure 10:**
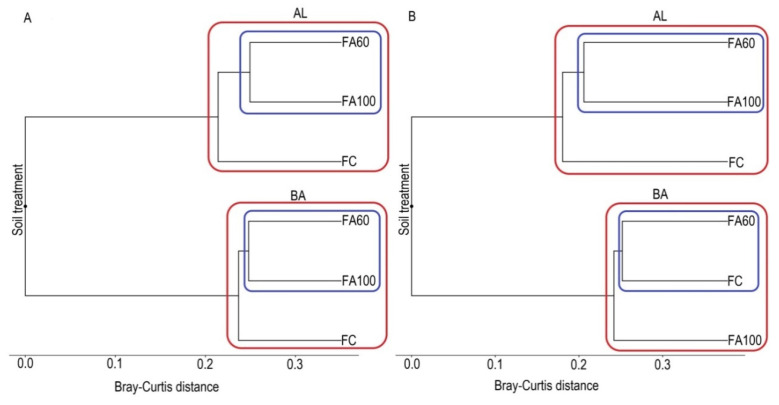
Cluster UPGMA dendrograms based on Bray-Curtis distances for soil bacterial (**A**) and fungal (**B**) communities. Explanation: FC—optimal dose of fertilizer, FA100—optimal dose of fertilizer enriched with microorganisms, FA60—fertilizer enriched with microorganisms (dose reduced by 40%), BA—Brunic Arenosol, AL—Abruptic Luvisol.

The Bray-Curtis dissimilarity distance was used for the construction of UPGMA dendrograms which indicate beta-diversity between the belowground microbial communities from the obtained soil treatments. The dendrogram showing the genetic relationships among the bacterial communities (Figure 10A) was based on a 16S rDNA nucleotide sequence analysis which indicated two main clusters which separately encompassed microorganisms inhabiting the AL and BA soil type, respectively. For both groups it was reported that bacteria from treatments with microbiologically enriched phosphorus mineral fertilizer formed individual clusters.

The UPGMA dendrogram for fungal communities (Figure 10B) is based on ITS1 nucleotide sequence analysis and showed two separated clusters. One group included the treatments from the AL soil type and second treatments from the BA soil type. A further division in the first group comprised fungal communities from the FA60 and FA100 treatments. For the second group it was reported that microorganisms from the FA60 and FC treatments formed a separate cluster.

**Figure 11:**
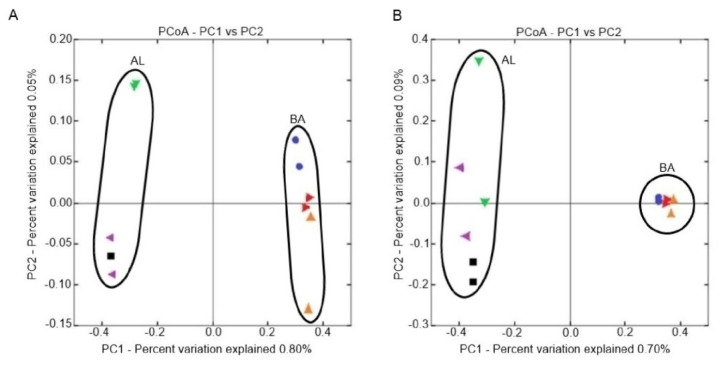
Principal coordinates analysis (PCoA) plots of the Bray-Curtis distances for soil bacterial (**A**) and fungal communities (**B**). Explanation: AL—Abruptic Luvisol, BA—Brunic Arenosol, green triangle—optimal dose of fertilizer AL, black square—optimal dose of fertilizer enriched with microorganisms AL, purple triangle-fertilizer enriched with microorganisms (dose reduced by 40%) AL, orange triangle—optimal dose of fertilizer BA, blue circle-optimal dose of fertilizer enriched with microorganisms BA, red triangle-fertilizer enriched with microorganisms (dose reduced by 40%) BA.

The principal coordinate analysis based on a Bray-Curtis dissimilarity showed evident clustering of treatments by soil type. Figure 11 indicates the distribution of beta-diversity between particular treatments. An analysis based on the 16S rDNA nucleotide sequence revealed two main clusters in which bacteria from the BA soil type are clearly separated from the bacterial communities in the AL soil type and the first two coordinates (PC1 and PC2) are explained, respectively, by an approximate 0.80% and 0.05% of the total variation in the bacteria communities (Figure 11A). For AL soil it was reported that the distribution of beta diversity had lower values between the FA100 and FA60 treatments when compared to the control soil. For the BA soil, beta diversity was higher within the controls in comparison with the FA100 and FA60 treatments.

The sequencing of the ITS1 region showed a similar trend as in the case of 16S rDNA analysis, namely two different clusters in the fungal metagenome were distinguished (Figure 11B). One group comprised fungal communities from the BA soil type and a second fungi from the AL soil. PC1 and PC2 coordinates explained, respectively, 0.70% and 0.09% of the total variation. For the BA soil, it was observed that beta diversity had approximate values across all treatments. For the AL soil, the differences between the beta diversity values were lower within treatments in which microbiologically enriched fertilizer was applied in comparison with FC treatments.

#### 2.4.3. Functional Prediction of the Bacterial Community

**Figure 12:**
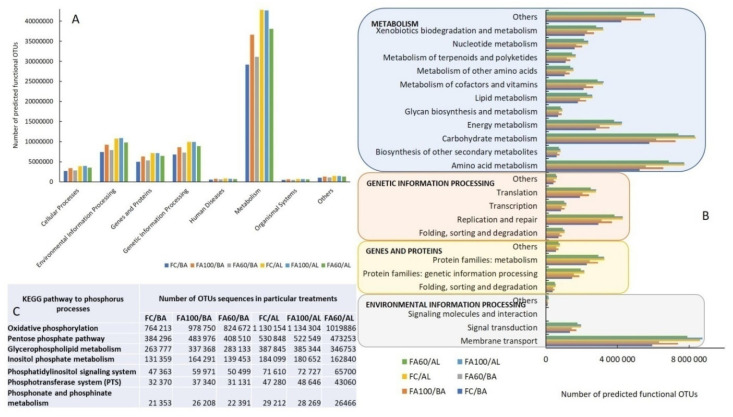
Variation in bacterial function profiles after biofertilizers application in Brunic Arenosol and Abruptic Luvisol soils analysed by PICRUSt. (**A**)—Biochemical metabolic pathways, (**B**)—Metabolism, (**C**)—Phosphorus processes based on KEGG function predictions. Explanation: BA—Brunic Arenosol, AL—Abruptic Luvisol, FC—optimal dose of fertilizer, FA100—optimal dose of fertilizer enriched with microorganisms, FA60—fertilizer enriched with microorganisms (dose reduced by 40%).

Bacterial function profiles predicted with the use of PICRUSt and based on the pathway database, KEGG, are presented in Figure 12. The majority of the predicted OTUs sequences annotated with the KEGG pathway in all tested treatments belonged to the following groups: metabolism group ~55%, environmental information processes ~14%, genetic information processes ~13%, genes and proteins ~9%, cellular processes ~5%, organismal systems ~1%, human diseases ~1% and other ~2%. For treatments with biofertilizers applied to BA soil a tendency to increase the sequences related to the main pathways group was observed, especially for treatment FA100. Such a tendency was not noted for AL soil (Figure 12A). Taking into account the results of the main KEGG classes, there were twelve pathways for metabolism, five for genetic information processing and four for genes and proteins and environmental information processing in this study (Figure 12B). In general, the number of sequences of particular KEGG classes were higher for AL in comparison with the BA soil type. However, for the BA soil the number of sequences of carbohydrate metabolism, amino acids metabolism, energy metabolism, lipid metabolism, xenobiotics biodegradation and metabolism increased for both treatments with biofertilizers (FA100, FA60) as compared to the control soil with mineral fertilization. A similar tendency was found for the sequences of translation, replication and repair, signal transduction and membrane transport. In contrast, for the AL soil type after the application of biofertilizers (FA100) only the sequences of membrane transport, energy metabolism and the metabolism of cofactors and vitamins increased, whereas the rest of the predicted functions generally decreased compared to the control soil (Figure 12B).

There were some differences in the efficiency of bacterial function connected with phosphorus processes among the tested treatments for the BA soil type. The abundance of sequences assigned to oxidative phosphorylation, the pentose phosphate pathway, glycerophospholipid metabolism, inositol phosphate metabolism, the phosphatidylinositol signalling system, the phosphotransferase system, phosphonate and phosphinate metabolism increased after biofertilizer application as compared to the control soil with mineral fertilization (Figure 12C). In contrast, the genes related to these pathways decreased or were at the same level as in the control soil for treatments tested in Abruptic Luvisol.

#### 2.4.4. Functional Guilds Prediction of the Fungal Community

**Figure 13:**
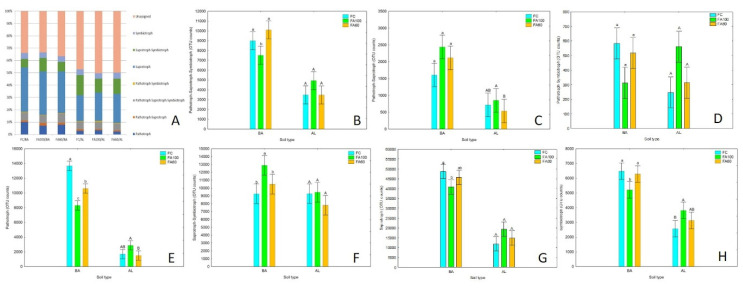
Relative abundance and OTUs counts for fungal functional groups (guilds) in two different soil types Brunic Arenosol and Abruptic Luvisol after biofertilizer application. (**A**)—fungal functional groups in tested soils and treatments, (**B**)—OTUs counts of pathotrophs-saprotrophs-symbiotrophs, (**C**)—OTUs counts of pathotrophs-saprotrophs, (**D**)—OTUs counts of pathotrophs-symbiotrophs, (**E**)—OTUs counts of pathotrophs, (**F**)—OTUs counts of saprotrophs-symbiotrophs, (**G**)—OTUs counts of saprotrophs, (**H**)—OTUs counts of symbiotrophs. Explanation: BA—Brunic Arenosol, AL—Abruptic Luvisol, FC—optimal dose of fertilizer, FA100—optimal dose of fertilizer enriched with microoganisms, FA60—fertilizer enriched with microorganisms (dose reduced by 40%). Vertical bars denote 0.95 confidence intervals. Different letters indicate significant differences (*p* < 0.05). The significant differences were calculated separately for each soil type by ANOVA and a post hoc analysis using the Tukey test (**B**,**E**,**F**,**H**) or Kruskal-Wallis and Dunn test (**C**,**D**,**G**). Different lowercase letters indicate significant differences within BA while uppercase letters within AL soil type.

Among the OTUs from the tested treatments of FC/BA, FA100/BA, FA60/BA, FC/AL, FA100/AL, FA60/AL, 65.98%, 66.53%, 66.44%, 52.69%, 49.68% and 50.22%, respectively were assigned to different functional groups, while the rest were unassigned. The functional groups found in all tested treatments included seven ecological guilds: pathotroph-saprotroph-symbiotroph, pathotroph-saprotroph, pathotroph-symbiotroph, pathotroph, saprotroph-symbiotroph, saprotroph, symbiotroph. The two ecological guilds saprotroph and saprotroph-symbiotroph were dominant for all tested treatments (Figure 13A). However, the OTUs counts of the saprotrophs, symbiotrophs and pathotrophs-saprotrophs in the Brunic Arenosol soil were approximately twice those identified in the Abruptic Luvisol soil (Figure 13C,G,H). Relative abundance and the total OTUs counts of pathotrophs in Brunic Arenosol soil after biofertilizers application (FA100, FA60) was significantly lower than those in the control soil (FC) (Figure 13A,E). No differences noteworthy in pathotrophs populations were found among the different treatments in Abruptic Luvisol (Figure 13E). However, a significantly higher proportion of symbiotrophs and pathotrophs-symbiotrophs in AL soil was identified for the FA100 treatment in comparison with the FC and FA60 treatments (Figure 13H,D).

The pathotroph population was mainly dominated by animal pathogens, which in general were present in a higher percentage in the control soils in comparison with treatments amended with biofertilizers and plant pathogens with higher counts in soils enriched with phosphorus biofertilizers (Table S5). The symbiotrophs were mainly dominated by ectomycorrhizal fungi, which showed an upward tendency after biofertilizers application for both doses (FA100, FA60) in Abruptic Luvisol and after FA100 incorporation in Brunic Arenosol. Finally, the saprotrophs consisted principally of unidentified saprotrophs fungi, which had a lower relative abundance and OTUs counts after biofertilizer use in Brunic Arenosol and a higher one in Abruptic Luvisol as compared to the control soil with the addition of only mineral fertilizers without microorganisms (Table S5).

## 3. Discussion

Different fertilization methods, both organic and inorganic, are known to influence the activity and biodiversity of soil microorganisms. The introduction of various substances to the soil may change the composition of indigenous microbiota and modify processes taking place in the soil environment [35,36,37].

At present, agricultural lands fertilized with biofertilizers fit into the growing trend of organic farming [29,38]. Biofertilizers were found to have a positive impact on multifarious aspects associated with agriculture. The application of beneficial microorganisms is known to improve nutrient exploitation, soil quality and fertility, plant growth, response to biotic and abiotic stresses and enhance crop yield [17]. It has also been documented that biofertilizers contribute to shifts in the biodiversity of soil microbiota [39], changes in soil biological and enzymes activities [40,41] and to the reduction in belowground pollutants [42]. Providing plants with nutrients which are not readily available is thought to be one of the most important functions of biofertilizers. Such elements include, among others, phosphorus whose availability in soil is limited and moreover, the excessive application of P mineral fertilizers may further diminish its uptake by plants. Therefore, it is important to implement microbial-based techniques that will improve the phosphorus availability and the efficiency of its uptake [43].

The activity of soil enzymes is thought to be an important indicator of soil quality and microbial community properties. Soil enzymes, produced by belowground microorganisms, are sensitive to various agronomic practices and changing environmental conditions, therefore an understanding of their role in soil functioning is necessary for the maintenance of an ecological balance in agricultural ecosystems [44,45]. In the presented study, we investigated the impact of phosphorus mineral fertilizer enriched with beneficial bacterial strains on the activity of four soil enzymes: β-glucosidase, protease, urease and acid phosphomonoesterase, these play a key role in substrates mineralization and in the biogeochemical cycles of carbon, nitrogen and phosphorus [46]. The obtained results showed that the application of biofertilizers did not adversely affected enzymes activity. For the treatments with applied biofertilizers an increase in enzymatic activity in relation to the control soil was noticeable. This indicates that the microorganisms contained in the fertilizers, in cooperation with indigenous microbiota, could metabolize compounds supplied to the soil more intensively, thereby providing nutrients for plant growth. The enhancement of enzymatic activity is of particular importance for FA60 treatments, because it presents the opportunity to reduce the dose of applied mineral fertilizer with a simultaneously advantageous effect on the intensity of biochemical processes occurring in the soil. The higher activity of β-glucosidase in FA100 and FA60 treatments, which has been proposed as an indicator of the soil organic matter (SOM) decomposition status, suggests an increased rate of SOM degradation [47]. As Lin et al. [48] noted, for soil amended with chemical fertilizers combined with organic manure, the activities of protease, urease, acid phosphatase and β-glucosidase remained at higher level as compared to the soil where only chemical fertilizers were used. The higher activity of β-glucosidade was also reported for soil in which wheat seeds were fertilized with the commercial products of Rhizosum N^®^ and Rhizosum PK^®^. Rhizosum PK^®^ is a biofertilizer containing i.a. *Bacillus megaterium* and *Frateuria aurantia* which were found to exhibit potassium-mobilizing properties [49,50,51]. A study conducted by Mengual et al. [52] showed that the introduction of microbial inoculants to the soil positively the affected activities of urease, protease and β-glucosidase with significant changes occurring in the case of urease and protease. The increased protease activity for FA100 and FA60 treatments may be associated with the capability of *Bacillus* sp. and *Paenibacillus* strains to produce heat stable proteases [53,54]. The limitations of phosphorus availability in soils are known to stimulate plant roots to secrete acid phosphatases which may contributed to an improvement in the aforementioned enzyme activity in AL soil in comparison with BA soil [55,56]. Proteases are enzymes that catalyze hydrolysis of peptide bonds in proteins, causing their breakdown into free amino acids [57]. In our study, increased protease activity was observed for the FA100 and FA60 treatments in both soil types, which may be associated with the increased availability of free amino acids in soils and their higher utilization rates in BIOLOG plates.

The functional diversity of soil microorganisms is another important parameter taken into account in assessing soil quality and fertility because it is connected with the activity of belowground microorganisms and their ability to adapt to soil environment modifications [58]. The BIOLOG system is widely used in environmental microbiology for the evaluation of the impact of various agricultural management practices [44,59], the contamination of soil and sewage sludge with heavy metals [60,61] and pesticides [62], the application of organic matter from organic wastes [63] and some stressing factors such as salinity, pH and heating [64] on the functional diversity of soil microbial communities.

The presented results of the metabolic profile of bacterial and growth intensity of fungal soil communities indicated differences in the functional diversity of soil microorganisms both between soil types and individual treatments. It is commonly known that, similarly to soil enzymatic activity, substrate use and the metabolic potential of soil microbes are an indicator of the intensity of biochemical processes occurring in the soil [65,66]. The highest level of metabolic diversity indices (AWCD, AWDD, H) were observed for the AL soil type which corresponds to increased enzymatic activities in the aforementioned soil type as compared to the BA soil type. The enhancement of the general metabolism of soil microorganisms for the FA100 and FA60 treatments is also indicated by the increased AWCD values [67].

An assessment of the ability of soil microorganisms to utilize different carbon sources is a rapid method for testing the differences and similarities between particular treatments [60]. In this work, supplying soil with microorganisms contained in biofertilizers contributed to shifts in the degree of utilization of C-compounds. Live microorganisms applied directly to the soil may influence the activity of indigenous soil microbiota [68] and changes in the level of utilization of some C-compounds reflect the changes to the metabolic abilities of microbial communities. With the simultaneous increase in the utilization of some *C*-substrates, a decrease in the use of others was noted, but their low level indicates the possibility of microorganisms’ adaptation to new conditions. According to Chaudhry et al. [69] higher functional diversity was observed for organically cultivated land as opposed to land management based on chemical fertilizers. The results obtained on ECO and FF plates showed that soil microorganisms are able to utilize various carbon sources. The increased utilization of particular polymers, amino acids, carbohydrates, carboxylic acids and amines for the FA100 and FA60 treatments suggest that the introduction of beneficial bacterial strains to the soil may enhance the metabolic properties of microorganisms towards certain compounds, which may constitute a source of essential nutrients, which are necessary not only for microorganisms but also for plants.

Through an analysis of utilization rates, it was reported that some amino acids and amines were metabolized more intensively in treatments with applied biofertilizers. Amino acids constitute not only a carbon, but also an important organic nitrogen source in soil [70]. The highest utilization rates of D-glucose-1-phosphate and D,L-α-glycerol phosphate, which may pose a phosphorus source for soil microbial communities, were also reported for AL soil. The increased utilization rate of the aforementioned compounds may be associated with the increased activity of microorganisms involved in the biochemical conversion of nitrogen and phosphorus. However, microorganisms more efficiently metabolize L-amino acids than their D-enantiomers [71] and our results suggest that the introduction of beneficial bacterial strains to the soil may accelerate processes responsible for L-amino acids utilization. These results are in accordance with the prediction results based on sequences obtained via NGS, where an increase in the sequences of amino acids metabolism under biofertilizers application into the soil was obtained.

It was observed that the utilization of polymers increased for the FA100 and FA60 treatments, as compared to the control treatment. According to Cheng et al. [72] Tween 80 was found to be easily biodegradable by soil indigenous microorganisms. It is also worth mentioning that microbial based techniques in combination with the addition of Tween 80 were successful in soil bioremediation from hydrophobic organic compounds. On the other hand, it was reported that the *Sphingomonas* strain in combination with Tween 80 was less effective in phenanthrene biodegradation. This may be explained by the fact that Tween 80 is the preferred carbon source over phenanthrene [73]. Moreover, a functional prediction of the bacterial community indicated that a higher number of sequences originated from xenobiotics biodegradation and metabolism in treatments with biofertilizers applied into BA soil and had no effect on AL soil, confirming that soil microbial community responses are dependent on soil type and properties.

It was described previously that carbohydrates comprise 70% of maize root exudates [74]. Considering the fact that oligo- and monosaccharides are preferred carbon source for microorganisms [75], it may be assumed that microbial strains inhabiting soil under maize cultivation have adapted their metabolic pathways in favour of carbohydrates utilization. The application of biofertilizers containing beneficial strains may stimulate indigenous microbiota to intensify the breakdown of carbohydrates. The increased metabolism of carbohydrates in FA100 and FA60 treatments may be connected with increased β-glucosidase activity in the aforementioned treatments [76]. Furthermore, the sequences related to carbohydrate metabolism increased for treatments with biofertilizers in the BA soil type.

Research concerning the diversity of soil microorganisms should also be based on an analysis of genetic diversity. In order to determine the structure of soil microorganisms, techniques based on the genetic fingerprint of the microbial communities are commonly used [77]. One of the most effective methods described in the literature is the analysis of terminal restriction fragment lengths polymorphism (t-RFLP) [78]. In this study a modified t-RFLP method was used, namely multiplex t-RFLP, which enables the simultaneous analysis of bacterial, archaeal and fungal soil microbial populations [79]. The obtained T-RFs patterns indicate changes in the genetic diversity after the application of biofertilizers. The presence of restriction fragments of the same length for all treatments may indicate the existence of bacterial, archaeal and fungal species which are commonly found, regardless of the environmental conditions. Changes observed in their relative abundance may result from shifts that occurred after the introduction of the biofertilizer. In the FA100 and FA60 treatments an increase in the T-RFs number was observed compared to the control soil. A similar trend was noticed by Trabelsi et al. [80], where soil inoculation, under potato cultivation, with rhizobial strains, contributed to an increase in the number of T-RFs in bacterial populations. Jaccard’s coefficient was analysed for each treatments and showed that microbial populations for the FA100 and FA60 treatments were more closely related to each other than to microorganisms in the control soil.

For a more detailed metataxonomic characterization of the soil microbial communities, next generation sequencing (NGS) was performed to identify representatives of different taxa present in soil amended with biofertilizers. The implementation of NGS in the field of environmental microbiology is very appealing for the exploration of soil microbial diversity due to the fact that it allows for an accurate and extensive analysis of multiple samples at the same time [81]. The metagenomic approach simplifies not only the study of phylogenetic relationships between microorganisms but it also determines their functionality in the soil environment [82].

Several studies have proven that fertilization strategies have an impact on the genetic diversity of soil microbial communities [83,84,85]. Our study revealed that the application of mineral fertilizer enriched with beneficial bacterial strains increased the number of 16S rDNA OTU’s in BA soil and ITS1 OTU’s for both soil types.

In the current study, Actinobacteria, Proteobacteria, Acidobacteria and Chloroflexi were the dominant phyla for all treatments in both soil types. Moreover, it was observed that for the FA100 and FA60 treatments, the relative abundance of Proteobacteria and Acidobacteria increased as compared with the control soil. Proteobacteria constitute the largest and the most diverse phylum in phylogenetic terms and along with Actinobacteria, their presence in the soil is connected with a high degree of carbon availability. The increased activity of β-glucosidase, which is involved in carbon cycling, in AL soil may be associated with the higher relative abundance of Actinobacteria and Proteobacteria in comparison with BA soil. At the same time, Acidobacteria is known to inhabit acidic and nutrient poor environments [86,87]. Generally, the members of the Proteobacteria and Acidobacteria group are commonly found in almost all soil types [88].

The results obtained with NGS showed that the relative abundance of potential nitrogen fixing strains increased for the FA100 and FA60 treatments, depending on soil type. The aforementioned nitrogen-fixing bacteria included Frankiaceae, Bradyrhizobiaceae and Rhodospirillaceae. Some of the members of the Frankiaceae group, especially those from the genus *Frankia* are known as a nitrogen-fixing symbiotic partners of actinorhizal plants [89]. The family of Bradyrhizobiaceae includes the genus *Bradyrhizobium*, which has members that are important microorganisms involved in biological nitrogen fixation (BNF) in legume plants. Due to possibility of using various nitrogen sources, bacteria from the Bradyrhizobiaceae family are vital constituents of the nitrogen cycle in the environment [90]. In considering the Rhodospirillaceae family, it is worth mentioning that one of the genus within Rhodospirillaceae is *Azospirillum*, which has representatives that exhibit plant growth promoting properties due to their N-fixing abilities [91].

Urease is an enzyme that catalyses the hydrolysis of urea to ammonia and carbon dioxide. Ammonia can then be oxidized to nitrates in the nitrification process, which may be conducted by ammonia oxidizing archaea (AOA) that belong to the Nitrososphaerales group in the Thaumarchaeota class [92]. Our results, which were obtained by both approaches NGS and t-RLFP, proved the occurrence of the aforementioned archaea in tested soils. The next step is denitrification and it has been described that many members of *Rhodoplanes* have the possibility of initiating this process [93]. *Rhodoplanes* lead to chemotrophic growth using denitrification in anoxic conditions in the presence of nitrates [94]. The increased urease activity for the FA100 treatments may be associated with an increase in the relative abundance of *Rhodoplanes* in the aforementioned treatments.

Members of the Syntrophobacteraceae family are described as sulfate-reducing bacteria [95]. In comparing the studied soils, the highest percentage share of this family in the bacterial community was reported in AL soil which may be associated with the fact that the experimental site was located near a sulphur mine.

The increased relative abundance of bacteria from the Actinomycetales order was reported for the FA100 and FA60 treatments in BA soil. Actinomycetales perform important functions in soil such as the decomposition of organic matter, suppressing some phytopathogens and the degradation of complex compounds in dead plants, animals and fungal manure. One noteworthy aspect of the bacteria is the fact that some Actinomycetales exhibit phosphorus solubilization and mineralization properties due to the production of organic acids and mechanisms encompassing chelation, exchange reactions and formation of polymeric compounds. It was also found that Actinomycetales secrete phosphatases, both acid and alkaline, and phytases, enzymes which are involved in belowground phosphorus biotransformations [10].

In the case of fungal communities, three phyla were identified with the domination of Ascomycota across all treatments in both soil types followed by Basidiomycota and Zygomycota. The high relative abundance of Ascomycota in both soil types may result from the fact that Ascomycota are the most numerous fungi in terrestrial environments [96].

In both soil types one of the dominant orders was Mortierellales and among them it was possible to distinguish some species: *Mortierellahorticola*, *Mortierellaelongata*, *Mortierella humilis* and *Mortierella* sp. However, the beneficial impact of *Mortierella* sp. strains on soil and plant properties has been described by researchers [97,98,99]. In this study it was reported that the relative abundance of some Mortierellales representatives increased in treatments fertilized with phosphorus biofertilizer. This result is in agreement with Li et al. [100] who observed that the relative abundance of *Mortierella* increased in soil treated with organic amendments. It is worth mentioning that, Li et al. [100] observed that the soil inoculated with *M. elongata* was characterized by a higher β-glucosidase activity. Another factor worth noting is the fact that some members of the *Mortierella* sp. are able to dissolve inorganic phosphorus compounds due to the production of oxalic acids [101,102] and to co-operate with mycorrhizal fungi in P uptake. Another strain, *M. humilis* was found to synthesize enzymes involved in the degradation of xylans, paraffin, chitin and some saccharides like cellulose, lignin, galactose, fructose and mannose [103]. Taking into account the beneficial properties of *M. elongata* described in the aforementioned study, the application of microbiologically enriched phosphorus mineral fertilizer seems to be a favourable option in improving the quality of the soil microbiome.

The Brunic Arenosol fungi from the *Penicillium* genus were characterized by quite a high relative abundance as compared to other identified fungi. The plant growth promoting properties of the *Penicillium* species include the production of antibiotics and plant hormones, protection from salinity and the induction of plant resistance. *Penicillium* also exhibit phosphate solubilization capabilities [104,105]. The most dominant *Penicillium* species was *P. simplicissimum* and it is worth noting that the relative abundance of this fungus increased for the FA60 treatment as compared to the control soil. According to Sangale et al. [106] *P. simplicissimum* is capable of degrading polyethylene and can induce resistance against Cucumber mosaic virus in tobacco plants and in *Arabidopsis thaliana* [107].

The fungi identified across all treatments for both soil types was *Fusarium oxysporum* which is considered to be one of the most widespread of the phytopathogenic fungi [108,109]. In this study it was reported that the relative abundance of *F. oxysporum* decreased for the FA60 treatment in AL soil and also for the FA100 treatment in BA soil as compared to the control treatments. The results from Qiu et al. [110] showed that the soil fertilized with amino acid fertilizer in combination with organic manure and strains of *Bacillus subtilis*, *Paenibacillus polymyxa* and *Trichoderma harzianum* was characterized by a lower relative abundance of *F. oxysporum* than soil devoid of the aforementioned beneficial microorganisms strains. According to Zhang et al. [111] *Paenibacillus polymyxa* is able to produce fusaricidin-type peptide antibiotics that suppress plant pathogenic fungi like *F. oxysporum*.

*Isaria fumosorosea* (formerly known as *Paecilomyces fumosoroseus*) is a fungi that appeared only in the FA100 treatment in the BA soil type at a relative abundance >1%, however in the FA60 treatment it was found that the relative abundance was higher than that found in the control soil. This microorganism is known as an entomopathogenic fungus and due to its various properties including a wide host range, the lack of a deleterious impact associated with the application of synthetic pesticides and the quality of remaining ecofriendly, it is thought to be a promising option as a biological agent against crop insects [112]. According to Kuźniar et al. [113] *Isaria fumosorosea* may be used as a bioinsecticide against the European corn borer (*Ostrinianubilalis* Hbn.) in sweet maize cultivation. Another identified enthomopathogenic fungus was *Metarhizium robertsii,* which was found to have a relative abundance that decreased in the FA100 and FA60 treatments in BA soil, however, it remained at a higher level in the FA60 than in the FA100 treatment. *Metarhizium* spp. strains were found to exert plant growth promoting properties encompassing the suppression of soil insects and plant pathogens, forming symbiotic relationships with plants and increasing nutrient uptake [114]. The results obtained from Sasan and Bidochka [115] showed that *Metarhizium robertsii* was able to establish close associations with the roots of switchgrass and haricot bean and to stimulate their growth and root hair proliferation.

The members of the Chaetothyriales sp., including the, *Cladophialophora* genus were identified in the BA soil type. These fungi are known as human pathogens, which may cause nervous system infections [116,117]. However, it was reported that in the FA100 and FA60 treatments the relative abundance of Chaetothyriales sp. decreased as compared to the control soil and, moreover, the relative abundance of *Cladophialophora* sp. in the FA100 and FA60 treatments remained below 1%. This may suggest that the beneficial strains contained in phosphorus biofertilizer exert antagonistic properties toward the Cladophialophora sp. species. A study conducted by Romero et al. [118] showed that the *Bacillus subtilis* strain may inhibit the growth of pathogenic fungus *Cladophialophora carrionii* in vitro.

NGS showed that the relative abundance of *Solicoccozyma terricola* increased in the FA60 treatment in BA soil as compared to the control. According to Stosiek et al. [119], the psychrotolerant strain *Solicoccozyma terricola* M 3.1.4. is capable of degrading glyphosate, which is commonly used in chemical pesticides; however, it was reported that glyphosate may pose a threat to living organisms. The application of the aforementioned fungus seems to be environmentally friendly tool in bioremediation and the introduction of beneficial microorganisms to the soil may positively affect the presence of other microbial strains useful in the field of ecology and agriculture.

The increased relative abundance of *Agrocybe pediades* and *Stropharia coronilla* was observed in the FA60 treatment and FA100 treatment in AL soil, respectively. These fungal strains are saprobic microorganisms [120,121] and, moreover, the strain of *Stropharia coronilla* was found to have the capability of metabolizing benzo[a]pyrene in a liquid culture [122].

Through cluster analysis, based on Bray-Curtis distances, we reported that the microbial communities from the FA100 and FA60 treatments were more similar to each other than those presented in the control soils. This suggests that the introduction of biofertilizers modified the composition of the microbial populations in both soil types. These results correspond to the results obtained with PCoA analysis, where a clustering of microorganisms from different soil types is clearly observed. This shows that for the tested treatments soil type and fertilization strategies are the main factors driving the structure of the microbial communities.

## 4. Materials and Methods

### 4.1. Study Site and Soil Sampling

Field experiments established in April 2018, were conducted on two different soil types, under maize cultivation (variety of P9241, FAO: K280, Z270, PIONEER, Warsaw, Poland). One field study was performed in Biszcza, South-East Poland (50°43′ N, 22°60′ E) on agricultural land that has been degraded due to inadequate cultivation and fertilization. The soil type was determined to be a Brunic Arenosol (BA) [41] with a pH_KCl_ of 4.8 and P, K, Mg contents of 174, 29 and 12 mg kg^−1^, respectively. The altitude of the study was about 211 m above sea level. Another field experiment was established in Basznia, South-East Poland (50°15′ N, 23°26′ E). The soil was classified as a Abruptic Luvisol (AL) [41] and it was chemically degraded as a result of the sulphur mine located near the experiment site. The soil pH_KCl_ at the beginning of the study was 4.9 and the contents of P, K, Mg were, respectively, 48, 53 and 36 mg kg^−1^. The experiment was located at an altitude of 230 m above sea level.

The doses of applied fertilizers were calculated based on the plant nutritional requirements and soil mineral content. The following mineral fertilizers were used in the study: phosphate mineral fertilizer SUPER FOS DAR 40 (Grupa Azoty, Puławy, Poland), nitrogen fertilizer PULREA PUŁAWSKI MOCZNIK 46N (Grupa Azoty, Puławy, Poland), granulated potassium salt (BIALCHEM, Poland). The microbial beneficial strains were provided by SYMBIOBANK and originated from the Research Institute of Horticulture in Skierniewice, Poland. In order to obtain biofertilizers, the phosphorus mineral fertilizer was enriched with *Paenibacillus polymyxa* strain CHT114AB, *Bacillus amyloliquefaciens* strain AF75BB and *Bacillus* sp. strain CZP4/4. The granules of fertilizer were coated with the mixture prepared in equal proportions 1:1:1 of each aforementioned strain [123]. The biofertilizers were prepared and provided by the Łukasiewicz Research Network–New Chemical Syntheses Institute (Puławy, Poland). A summary of the treatments that were conducted within both experiments is presented in Table 3.

Because of that much of the P applied to the soil as mineral fertilizers is bound to the soil, thereby creating a pool of residual P, or it is lost through leaching, runoff, or erosion and may contribute to the eutrophication of waterbodies [124], the research carried out included the application of a 40% reduced dose of phosphorus fertilizer enriched with beneficial bacteria strains which have the potential to activate phosphorus present in the soil. Each type of fertilization was arranged in 3 replications with 10 m × 15 m plots for both soil types. The soil sampling in this study was performed in June 2018, one week after fertilizer application. The soil samples were collected from the 0–25 cm layer from five sites within each plot and then averaged by homogenization and intensive mixing. The samples were transported afterwards under refrigeration at 4 °C to the laboratory and sieved through a 2 mm sieve. The soil samples were promptly used for measurements or stored (at 4 °C for CLPP and enzymatic activity or at −80 °C for DNA extraction).

### 4.2. Enzymatic Activities

Urease activity was determined according to the Zantua and Bremner [125] method with the use of a urea solution as a substrate, after 18-h soil incubation at 37 °C. The wavelength of urease activity was measured at 410 nm.

Protease activity was assessed using the Ladd and Butler [126] method as a modification of Alef and Nannipieri [127], after one hour of soil incubation at 50 °C with a Tris-HCl (pH 8.1) sodium caseinate solution as a substrate. The concentration of released tyrosine was measured spectrophotometrically at 578 nm.

β-Glucosidase activity was determined using the Eivazi and Tabatabai [128] method modified by Alef and Nannipieri [127]. This method is based on the determination of the p-nitrophenol (PNP) released after the incubation of soil with a p-nitrophenyl-*β*-d-glucoside (PNG) solution as a substrate, for 1 h at 37 °C. The enzymatic activity was measured colorimetrically at a wavelength of 400 nm.

The acid phosphomonoesterase activity was assessed according to the Tabatabai and Bremner method [129] after one-hour of soil incubation at 37 °C using a PNP in Tris-HCl buffer at pH 6.5. The concentration of released PNP was determined spectrophotometrically at a wavelength of 400 nm.

The results were calculated with reference to the oven-dry (105 °C) weight of soil.

### 4.3. Community Level Physiological Profiling (CLPP)

One way to determine functional diversity is community level physiological profiling (CLPP) with the application of plates (BIOLOG^®^, Hayward, CA, USA) coated with various carbon sources and dedicated to specific groups of microorganisms. As a result, a characteristic and unique utilization pattern of the C-substrates called “metabolic fingerprinting” is obtained [130,131]. For the evaluation of the catabolic abilities of soil bacterial and fungal communities, BIOLOG ECO and BIOLOG FF microplates were used, respectively [132]. The ECO microplate consists of a set of 31 different carbon sources plus a water control, in three replications, contained in 96-well microtiter plates [133]. The BIOLOG FF microplate consists of 95 various carbon sources and a non-C control distributed in 96-well microtiter plates [134].

The CLPP analysis using BIOLOG plates was prepared in the following way: 1 g of fresh soil was suspended in 99 mL of sterile saline peptone water and shaken for 20 min at 20 °C before being incubated for 30 min at 4 °C. Afterwards, each well of the BIOLOG ECO plate and FF plate was inoculated with a prepared suspension of 120 µL and 100 µL respectively, and incubated at 23 °C. Absorbance readings were performed at 590 nm in the case of ECO plates and at 750 nm as for FF plates with a BIOLOG MicroStation plate reader (Biolog^®^, Hayward, CA, USA) every 24 h for 216 incubation hours. Detailed procedures of the analyses were described by Wolińska et al. [135] and Gryta et al. [35].

### 4.4. DNA Extraction

Genomic DNA was extracted from 0.5 g of fresh soil samples using a FastDNA Spin Kit for Feces (MP Biomedicals, Solon, OH, USA) according to manufacturer protocol. The isolated DNA was then eluted in 50 µL of TES buffer. The quantity of extracted DNA was determined spectrophotometrically at a wavelength of 260 nm and the purity was determined using coefficients (260/230, 260/280) calculated on the basis of absorbance readings at 230, 260 and 280 nm (NanoDrop 2000/2000c, Thermo Scientific, West Palm Beach, FL, USA). The extracted DNA was then stored at −20 °C for further analyses comprising multiplex terminal restriction fragments length polymorphism (M-tRFLP) and next generation sequencing (NGS).

### 4.5. Multiplex Terminal Restriction Fragment Length Polymorphism (M-tRFLP)

In this work, the multiplex t-RFLP (M-tRFLP) fingerprinting method was used, which allowed for a simultaneous analysis of three microbial taxa: bacteria, archaea and fungi. The M-tRFLP reaction includes: DNA amplification with fluorescently labelled individual taxon-specific primers, digestion using restriction endonuclease selected appropriately for all amplification products present in the reaction mixture and the separation of the restriction products using a genetic analyser [136].

The first step of the M-tRFLP analysis was the PCR reaction for all extracted DNA samples. For the bacterial community analysis, the PCR primer pair (63f/1087r) targets the 16S rDNA region was used and the primers were as follows: primer F: 5′-AGGCCTAACACATGCAAGTC-3′ [137] and primer R:5′-HEX-CTCGTTGCGGGACTTACCCC-3′ [79,138]. For the fungal community analyses, the PCR primers (ITS1F/ITS4R) were used to amplify the ITS1 spacer and the primers had the following sequences: primer F: 5′-6-FAM-CTTGGTCATTTAGAGGAAGTAA-3′ [139] and primer R: 5′-TCCTCCGCTTATTGATATGC-3′ [140]. For the archaeal community analyses the PCR primer pair (Ar3F/Ar9R) was used and the primers were as follows: primer F: 5′-TTCCGGTTGATCCTGCCGGA-3′ [141] and primer R: 5′-ROX-CCCGCCAATTCCTTTAAGTTTC-3′ [79,142]. Each PCR reaction mixture contained 15 µL of RedTaq^®^ReadyMix™ PCR Reaction Mix (Sigma-Aldrich, St. Louis, MO, USA), 4 ng of genomic DNA, in the case of the bacterial mixture: 0.5 µLwas used and for the fungal and archaeal mixture: 1 µL each of the forward and reverse primers (diluted to a final concentration 10 µM) were used. The PCR reaction was performed with a gradient thermal cycler (Veriti 96 well Fast Thermal Cycler) with the following temperature cycle: 95 °C initial denaturation for 5 min, followed by 30 cycles at 95 °C denaturation for 30 s; 55 °C annealing for 30 s; 72 °C elongation for 60 s, and a final extension at 72 °C for 10 min. The presence and size of the amplification products (bacteria 1000 bp, archaea 900 bp and fungi 600 bp) were checked electrophoretically (110 V, 40 min) on a 2% agarose gel, which was stained with a SimplySafe (EURx, Gdańsk, Poland) solution and visualized with UV excitation. Subsequently, the PCR products were purified by using a mixture of thermo sensitive alkaline phosphatase and Exonuclease I (Exo-BAP-Mix, EURx) followed by incubation at 37 °C for 15 min and then at 80 °C for 15 min. After the Exo-BAP reaction, the amplification products were purified with Performa^®^ DTR (Dye Terminator Removal) Gel Filtration Cartridges (EdgeBio, San Jose, CA, USA) according to the producer protocol. The amount and quality of DNA was determined by a spectrophotometer at a wavelength of 260 nm. The purified PCR products were then digested by endonuclease HaeIII (EURx, Gdańsk, Poland). The restriction mixture (13 µL) containing 5–10 µL of purified PCR products (about 50 ng of DNA), 0.6 µL of restriction enzyme (10 U/µL), 0.6 µL of ONE buffer containing bovine serum albumin (BSA) (EURx, Gdańsk, Poland) and water, was incubated at 37 °C for 2 h. Enzyme inactivation was performed by incubation at 80 °C for 20 min. Aliquots (1 µL) of the digest were mixed with 9 µL of deionized formamide and 0.5 µL of DNA fragment length standard GS-600LIZ (Applied Biosystems, Foster City, CA, USA). The restriction samples were applied on the plate in three replications for each sample. Then, the plate was denaturated at 94 °C for 3 min and chilled on ice. The sizes of the fluorescently labelled restriction fragments were determined by capillary electrophoresis using an automated ABI 3130 Genetic Analyzer (Applied Biosystems, Foster City, CA, USA). The procedure was optimized and described in detail by Gryta and Frąc [79]. The obtained results were analyzed using GeneMapper v. 4.0. software (Applied Biosystems, Foster City, CA, USA). The analysis included restriction fragments larger than 50 bp and >1% of the total area within the sample. Based on in silico analysis using TRiFLe software [32] the terminal restriction fragments (t-RFs) were identified using The National Center for Biotechnology Information (NCBI) database (https://www.ncbi.nlm.nih.gov/) [33] for 16S rDNA and ITS1 rDNA and *amo*A AOA Feifei-Liu reference database from the FunGene functional gene pipeline and repository [143].

### 4.6. Next Generation Sequencing (NGS)

Next generation sequencing was conducted at Genomed S.A. (Warsaw, Poland) on Illumina MiSeq platform (Illumina Inc., San Diego, CA, USA). A metataxonomic analyses of the prokaryotic (bacterial and archaeal) and eukaryotic (fungal) communities were performed based on the hypervariable region V3–V4 of the 16S rDNA gene and hypervariable region ITS1, respectively. The MiSeq platform (Illumina Inc., San Diego, CA, USA) was used to sequence the DNA of the microorganisms. The following primers: 341F (5′-TCG TCG GCA GCG TCA GAT GTG TAT AAG AGA CAG CCT ACG GGN GGC WGC AG-3′) and 785R (5′-GTC TCG TGG GCT CGG AGA TGT GTA TAA GAG ACA GGA CTA CHV GGG TAT CTA ATC C-3′) were used for the V3-V4 region of 16S rDNA [144]. Whereas, the primers ITS1FI2 (5′-TCG TCG GCA GCG TCA GAT GTG TAT AAG AGA CAG GAA CCW GCG GAR GGA TCA-3′) and 5.8S (5′-GTC TCG TGG GCT CGG AGA TGT GTA TAA GAG ACA GCG CTG CGT TCT TCA TCG-3′) were used to amplify the ITS1 rDNA region [145,146]. In order to perform an amplification and prepare the libraries, the above-mentioned primers and Q5 Hot Start High-Fidelity 2× Master Mix were applied as recommended by the manufacturer (NEB Inc., Ipswich, MA, USA). The v2 Illumina kit was applied for sequencing using 2 × 250 bp pair-ended technology.

### 4.7. Statistical and Bioinformatics Analyses

All statistical analyses were performed with Statistica 13.1 software (StatSoft Inc., Tulsa, OK, USA). The *C*-compounds utilization patterns were prepared using heatmap graphs. The enzyme activities, Average Well Color Development (AWCD) [147], Average Well Density Development (AWDD) [148], Richness (the number of utilized carbon sources), number of OTU’s (operational taxonomic units) and Shannon index (H) [149] differences between the treatments were assessed with appropriate statistical tests. The analysis of variance (ANOVA) regarding the soil type and applied fertilization and a post-hoc Tukey test were used for significant differences calculation when ANOVA assumptions were met. To verify whether ANOVA assumptions, including dataset normality and homoscedasticity of the variance were met, Shapiro-Wilk and by Levene tests were used, respectively. If normality assumption of a parametric test was violated, then Kruskal-Wallis and Dunn test was used instead. If normality of dataset was met but homoscedasticity of variance was violated, then F-Welch test with post hoc Tukey test was used instead.

Beta diversity between treatments was measured using Bray-Curtis distances and visualized in the form of UPGMA dendrograms and with the application of principal coordinate analysis (PCoA).

The Venn diagrams were constructed with the application of the Venny 2.1 online program [150].

Jaccard’s coefficient index was calculated using the following formula:

(1) $$Jaccard\;similarity\;between\;profile\;A\;and\;B\;=\frac{N_{AB}}{N_{A}+N_{B}-N_{AB}}$$

where *N_AB_*_—_number of common T-RFs present in both profile *A* and *B*, *N_A_*—number of T-RFs in profile *A*, *N_B_*—number of T-RFs in profile *B* [151].

MiSeq Reporter (MSR) v. 2.6. software (Illumina Inc., San Diego, CA, USA) was used for the first preliminary processing of the data, including demultiplexing and the generation of fastq files. The Quantitative Insights into Microbial Ecology (QIIME) tool was used to elaborate the raw sequence reads [152]. The taxonomical classification of 16S V3–V4 OTUs was achieved using the uCLUST algorithm [153] and the GreenGenes v. 13_8 database [154], while a Basic Local Alignment Search (BLAST) [155] against the UNITE database [156] was used for the ITS1 region [157].

In order to predict the functional responses to the application of biofertilizers to two different soil types, PICRUSt (Phylogenetic Investigation of Communities by Reconstruction of Unobserved States, software) [158] was used to generate a functional profile from the 16S rDNA data. Prior to the metagenomes prediction, the OTUs of the 16SrDNA sequences were normalized using PICRUSt. PICRUSt and Kyoto Encyclopedia of Genes and Genomes (KEGG) [159] were used to prepare classes of functional genes according to the KEGG module, which were present in particular soil samples. Moreover, the FUNGuild online application was used to assign functional information including the ecological guild of fungi [160] to OTUs obtained in high-throughput sequencing datasets based on ITS1 rDNA.

## 5. Conclusions

In summary, we have analysed the impact of adding phosphorus mineral fertilizer enriched with strains of beneficial microorganisms on the soil microbiome status. The study encompassed genetic and functional diversity and enzymatic activity. The introduction of live microorganisms to the soil enhanced the activity of the soil enzymes, modified metabolic pathways toward certain compounds and increased genetic diversity. It is noteworthy that no negative shifts in the microbial communities (based on analysing the relevant parameters) were observed after the application of biofertilizers. Of particular interest are the changes activated by introducing into the soil a reduced dose of mineral fertilizer, but microbiologically enriched. This creates the opportunity of limiting the doses of mineral fertilizers used in agriculture with a simultaneous beneficial impact on the environment. The results presented in this study show that biofertilization promotes the occurrence of microbial strains involved in the circulation of essential nutrients, the decomposition of organic matter and the eradication of potentially pathogenic organisms is also promoted. This is a very important effect, taking into account that the aforementioned properties contribute to the improvement of soil fertility and quality and, eventually to improved crop yields. A variation in bacterial function profiles after biofertilizer application was analyzed using predictive functional profiling of microbial communities based on 16S rDNA marker gene sequences which indicated that multiple metabolic pathways occurred in two different soil types. Biofertilizer application shifted the composition and functional ecological guilds in the soil fungal communities. In general, there was an upward tendency in the abundance of ectomycorrhizal fungi which dominated the symbiotrophs group after biofertilizers application. In contrast, there was a significant decrease in the relative abundance of pathotrophs in Brunic Arenosol soil after biofertilizer application, but no remarkable differences in pathotrophs were found among the different treatments in Abruptic Luvisol. Our work suggests that mineral fertilizers enriched with beneficial microbial strains may be an alternative to traditional chemical fertilizers leading to a reduction in the consumption of mineral fertilizers, which is in accordance with the latest policy initiatives and law regulation, such as The European Green Dealand EU Biodiversity Strategy for 2030. However, soil type and properties should be taken into consideration during biofertilizers use, due to the various responses of the tested microbial communities in different soils.

## Figures and Tables

**Table 1:** Average or median values of AWCD, AWDD, H, R indices and number of OTUs. Different letters indicate significant differences (*p* < 0.05) calculated separately for each soil type by ANOVA post hoc Tukey test (AWCD, ECO Plates R, AWDD, FF Plates R, NGS 16S H, t-RFLP archaea R, t-RFLP archaea H), F-Welch and post hoc Tukey test (ECO Plates H, FF Plates H) or Kruskal-Wallis and Dunn test (NGS 16S OTU, NGS ITS1 OTU, NGS ITS1 H, t-RFLP bacteria R, t-RFLP bacteria H, t-RFLP fungi R, t-RFLP fungi H).

Soil Type/Treatment							
		BA			AL		
		FC	FA100	FA60	FC	FA100	FA60
**ECO Plates**	AWCD *	0.81 ± 0.08 a	0.70 ± 0.16 b	0.80 ± 0.03 ab	0.81 ± 0.32 a	0.91 ± 0.06 a	0.92 ± 0.13 a
	R *	20.00 ± 1.00 a	19.00 ± 3.00 a	19.00 ± 2.00 a	21.00 ± 3.00 a	21.00 ± 1.00 a	22.00 ± 0.00 a
	H *	2.84 ± 0.07 a	2.87 ± 0.07 a	2.74 ± 0.12 a	3.05 ± 0.11 a	2.92 ± 0.29 a	3.04 ± 0.02 a
**FF Plates**	AWDD *	0.55 ± 0.25 a	0.60 ± 0.09 a	0.51 ± 0.11 a	0.40 ± 0.17 a	0.47 ± 0.08 a	0.38 ± 0.06 a
	R *	52.00 ± 13.00 a	58.00 ± 9.00 a	56.00 ± 10.00 a	52.00 ± 13.00 a	58.00 ± 4.00 a	51.00 ± 6.00 a
	H *	3.82 ± 0.41 a	3.92 ± 0.16 a	3.88 ± 0.13 a	3.88 ± 0.14 a	3.94 ± 0.10 a	3.83 ± 0.15 a
**NGS 16S**	OUT **	2549 min 2507; max 2590 a	2744 min 2736; max 2750 a	2578 min 2565; max 2590 a	3037 min 2998; max 3976 a	2928 min 2925; max 2930 a	3038 min 3035; max 3040 a
	H *	8.78 ± 0.01 a	8.94 ± 0.01 a	8.79 ± 0.01 a	9.14 ± 0.02 a	9.12 ± 0.01 a	9.19 ± 0.01 a
**NGS ITS1**	OUT **	1234 min 1215; max 1251 a	1342 min 1325; max 1358 a	1338 min 1329; max 1345 a	1385 min 1296; max 1471 a	1411 min 1351; max 1470 a	1441 min 1424; max 1458 a
	H **	6.47 min 6.38; max 6.56 b	6.83 min 6.83; max 6.84 a	6.75 min 6.74; max 6.75 ab	7.23 min 7.16; max 7.30 a	7.51 min 7.45; max 7.57 a	7.61 min 7.50; max 7.71 a
**t-RFLP bacteria**	R **	3.00 min 2.96; max 3.00 b	7.00 min 4.98; max 8.96 ab	10.00 min 9.98; max 10.00 a	8.00 min 6.99; max 8.00 a	11.00 min 10.99; max 11.00 a	9.00 min 6.65; max 10.98 a
	H **	1.04 min 1.03; max 1.04 b	1.86 min 1.59; max 2.13 ab	2.21 min 2.20; max 2.22 a	1.86 min 1.84; max 1.87 a	2.02 min 2.00; max 2.04 a	1.91 min 1.72; max 2.09 a
**t-RFLP archaea**	R *	24.00 ± 1.00 a	24.00 ± 0.00 a	23.00 ± 2.00 a	26.00 ± 4.00 a	25.00 ± 3.00 a	30.00 ± 3.00 a
	H *	3.01 ± 0.03 ab	3.04 ± 0.00 a	2.93 ± 0.08 b	3.16 ± 0.17 a	3.10 ± 0.19 a	3.33 ± 0.04 a
**t-RFLP fungi**	R **	11.00 min 9.97; max 11.99 ab	16.00 min 15.98; max 16.00 a	10.00 min 7.99; max 11.01 b	19.00 min 11.96; max 26.00 a	25.00 min 19.90; max 28.93 a	29.00 min 26.98; max 29.92 a
	H **	2.26 min 2.20; max 2.32 a	2.62 min 2.58; max 2.65 a	2.11 min 1.88; max 2.32 a	2.69 min 2.16; max 3.20 a	2.93 min 2.71; max 3.15 a	3.15 min 3.11; max 3.17 a

Explanation: ±—standard deviation, min—minimal value, max—maximal value, BA—Brunic Arenosol, AL—Abruptic Luvisol, FC—optimal dose of fertilizer, FA100—optimal dose of fertilizer enriched with microorganisms, FA60—fertilizer enriched with microorganisms (dose reduced by 40%), AWCD—Average Well Color Development, AWDD—Average Well Density Development, R—Richness, H—Shannon index, OUT—operational taxonomic units, *—average values, **—median values.

**Table 2:** Jaccard’s coefficient values *. Explanation: FC—optimal dose of fertilizer, FA100—optimal dose of fertilizer enriched with microorganisms, FA60—fertilizer enriched with microorganisms (dose reduced by 40%).

	Soil Type					
	BrunicArenosol (BA)			Abruptic Luvisol (AL)		
Treatment	Bacteria	Archaea	Fungi	Bacteria	Archaea	Fungi
FC-FA100	0.33	1	0.81	0.64	0.97	0.84
FC-FA60	0.30	1	0.61	0.62	0.94	0.84
FA100-FA60	0.90	1	0.75	0.85	0.97	1

* Jaccard’s coefficient was calculated based on the number of peaks; the peak was taken into account when was observed in at least two out of three replicates; no differences in the number of peaks between replicates were observed.

**Table 3:** The treatments arrangements within the field experiments. The doses of applied; fertilizers/biofertilizers were calculated per ha separate for each soil type.

Treatment	Brunic Arenosol (BA)	Abruptic Luvisol (AL)
Optimal dose (FC)	125 kg phosphate mineral fertilizer; 365 kg urea; 290 potassium salt	150 kg phosphate mineral fertilizer; 360 kg urea; 284 kg potassium salt
Optimal dose enriched with microorganisms (FA100)	156.25 kg phosphate mineral fertilizer enriched with beneficial bacterial strains; 365 kg urea; 290 potassium salt	187.5 kg phosphate mineral fertilizer enriched with beneficial bacterial strains; 360 kg urea; 284 kg potassium salt
Dose reduced by 40% enriched with microorganisms (FA60)	93.75 kg phosphate mineral fertilizer enriched with beneficial strains; 365 kg urea; 290 potassium salt	112.5 kg phosphate mineral fertilizer enriched with beneficial bacterial strains; 360 kg urea; 284 kg potassium salt

## Supplementary Materials

Supplementary materials can be found at https://www.mdpi.com/1422-0067/21/21/8003/s1.
